# Supplementary material for: Absence of High Lipoprotein(a) Levels Is an Independent Predictor of Acute Myocardial Infarction without Coronary Lesions
Source: J Clin Med. 2023 Jan 26;12(3):960. doi: 10.3390/jcm12030960 (PMC9917543; doi:10.3390/jcm12030960)
Supplement: Supplementary file 1 [file jcm-12-00960-s001.zip › jcm-2139810-supplementary.pdf]

Supplemental Table S1. Cox proportional hazard model for the incidence rates of acute ischemic events and death.

		Crude	
**Parameter**		**HR (95% CI)**	***p***
No lesions		0.67 (0.39–1.18)	0.174
Cardiovascular Event (STEMI)		1.30 (0.99–1.71)	0.057
Sex (Male)		0.90 (0.66–1.22)	0.486
Age (years)		1.03 (1.02–1.05)	<0.001
Race (Caucasian)		1.26 (0.47–3.39)	0.647
Estimated Glomerular Filtration Rate		0.98 (0.97–0.99)	<0.001
Smoker		0.73 (0.55–0.97)	0.030
Diabetes		1.77 (1.32–2.37)	<0.001
Hypertension		1.98 (1.47–2.68)	<0.001
Dyslipidemia		1.08 (0.82–1.43)	0.572
Previous Myocardial Infarction		1.94 (1.37–2.74)	<0.001
Peripheral Artery Disease		1.80 (1.11–2.92)	0.017
Cerebrovascular Accident		2.35 (1.39–3.98)	0.001
Atrial Fibrillation		2.16 (1.45–3.21)	<0.001
Previous Heart Failure		2.15 (1.54–3.01)	<0.001
Coronary Artery Bypass Grafting		3.20 (1.94–5.26)	<0.001
Body Mass Index		1.01 (0.98–1.04)	0.447
Familiar background		1.06 (0.75–1.50)	0.741
Eating Fruit		0.97 (0.90–1.05)	0.502
Eating Fish		1.07 (0.98–1.16)	0.120
Alcohol consumption	0	Ref.	-
	1–7	0.70 (0.50–1.02)	0.067
	8–14	0.93 (0.62–1.39)	0.709
	>14	0.78 (0.49–1.25)	0.305
Type of revascularization	No revascularization	Ref.	-
	Loaded stent	1.32 (0.61–2.82)	0.480
	Conventional stent	1.16 (0.56–2.37)	0.694
	Angioplasty	1.14 (0.54–2.39)	0.731
	Coronary artery bypass graft	1.16 (0.40–3.36)	0.778
Ejection fraction <40%		1.25 (0.88–1.79)	0.222
Hs Troponin		1.00 (1.00–1.00)	0.835
LDL-c		1.00 (1.00–1.00)	0.037
Triglycerides		1.00 (1.00–1.00)	0.257
HDL-c		0.99 (0.98–1.01)	0.342
IL-18		1.00 (1.00–1.00)	0.768
PCSK9		1.00 (1.00–1.00)	0.738
Lp(a) > 60 mg/dL		1.60 (1.14–2.23)	0.006
Hs-CRP		1.00 (0.99–1.01)	0.935
Medical Therapy at Discharge			
Acetylsalicylic acid		0.46 (0.28–0.77)	0.003
AntiP2Y12		0.80 (0.53–1.20)	0.280
Acenocumarol		2.21 (1.46–3.33)	<0.001
Statins		0.45 (0.27–0.75)	0.002
Ezetimibe		1.94 (0.86–4.37)	0.111
Insulin		2.62 (1.76–3.91)	<0.001

Oral antidiabetic drugs	1.49 (1.06–2.09)	0.023
ACE inhibitors	0.85 (0.61–1.18)	0.339
Aldosterone receptor blockers	1.41 (0.91–2.20)	0.125
Betablockers	0.81 (0.58–1.14)	0.233
Nitrates	2.03 (1.47–2.80)	<0.001
Diltiazem	1.77 (0.91–3.46)	0.094
Dihydropyridines	1.94 (1.33–2.83)	0.001
Diuretics	2.19 (1.60–2.99)	<0.001
Proton Pump Inhibitors	0.98 (0.72–1.35)	0.915
Digoxin	5.66 (1.40–22.8)	0.015
Amiodarone	0.83 (0.31–2.23)	0.71

Abbreviations as for Table 1.
